# Turning over New Leaves

**Published:** 1888-11-10

**Authors:** 


### TURNING OVER NEW LEAVES.

### Self-Help a Hundred Years Ago.*

WITH regard to style and arrangement of matter, Mr. Holyoake's book belongs to the "sheep's head" order of literature —"there is a grand lot of miscellaneous reading in it," without much attention to strict sequence of either time or thought. But the matter is decidedly worth seeking and preserving, though it contains something of a snub to this conceited generation, which is rather of opinion that it has invented the science of thoughtful and well-considered philanthropy. Certainly there has within recent years been a strong reaction against that doctrine of the eternal fitness of that struggle for existence ending, as a rule, in the survival of the most unscrupulous and selfish, which finds its expression in Benthamism in the field of political economy, and in Darwinism in that of science. But the books, letters, and pamphlets which Mr. Holyoake has unearthed with infinite labour and from many hiding-places, prove that at the end of the last and the beginning of the present century, when as yet the Evolution theory was not, there lived men who thought it their duty to help their poorer neighbours to help themselves.

Very curious information do we get in this volume—for example, that the first co-operative store was founded in the village of Mongewell, in the county of Oxford, in the year 1794, by the Bishop of Durham, whose example was followed by the Rev. Dr. Glasse, of Greenford, a hamlet within a mile of Harrow-on-the-Hill. Dr. Glasse was less broad-minded than Bishop Barrington, and insisted that those of his parishioners who wished to profit by the better quality and smaller prices of the provisions in the store must earn the privilege by regular attendance at church. He gained his point by issuing tickets admitting to the advantages of the store for a week, which were delivered to applicants by the parish clerk after morning or evening service on Sunday. He flattered himself that thereby he promoted the spiritual as well as the temporal welfare of his flock; but it may be doubted if the experiment did a great deal for true religion. Henry of Navarre did not become a model Catholic because he said "*Paris vant bien une messe*," and perhaps the Greenford peasants merely thought, after the same fashion, that a piece of good bacon was worth a sermon.

It will be news to many that the war-cry of "three acres and a cow," which Mr. Jesse Collings is credited with originating, first arose about ninety years ago; and it should be noted that evidence came from several different localities to the effect that in those parishes where labourers had these small allotments they remained self-supporting in old age, and the poor-rate remained low even when it was raised in every district around. Those who think that the labourer's cow is a disguised pioneer of Communism should be reassured by learning that the Earl of Winchilsea said, in 1798, with regard to the necessary pasture: "No rents are better or more regularly paid on my estate than those for the cottagers' lands. There has not been for several years back any arrears of them." Sir Thomas Bernard also, in editing the paper in which Lord Winchilsea narrates his experiences, makes the following wise reflection:

"Viewed in a political light, the labourer who has property, however small—a cow, a pig, or even the crop of his garden —has an interest in the welfare and tranquility of his country, and in the good order of Society. He who has no property is always ready for novelty and experiment; and though gibbets and halters may for a time deter him from criminal and atrocious acts, yet no motive exists to fix him in virtuous habits, or to attach him to that national prosperity in which he has no part, and to that constituted order of property which excludes him from all possession."

A most interesting account is given of the prison at Dorchester, which, in 1791, was developed into an industrial establishment in the case of all prisoners who were either compelled by law or could by encouragement be compelled to work. The most notable feature of the scheme was that the prisoners were allowed a certain proportion of profit from their work. "Convicts, and all persons sentenced to imprisonment and hard labour, are allowed one-sixth of their earnings, besides broth in addition to their bread; and if they earn to the amount of five shillings a-week they are also allowed meat. The full amount, however, of the shares of the earnings, except those of debtors, are not paid to the prisoners until their discharge, but are carried to their respective accounts, and twopence a-week only is allowed them for the purpose of procuring for themselves any little indulgences. There have been instances of men who have received eight or ten pounds, or more, on quitting the prison, and the money has, for the most part, been laid out by them in clothes, tools, furniture, a stock of bacon or other provisions, etc., for their future comfort and advantage."

The result of this system was that the prisoners left the gaol with their characters improved by the habit of steady, honest work, and with the means in their hand, provided by their own industry, of seeking a continuance in it. Surely this is better than cranks and tread-mills.

Among the other questions treated of in this volume are the mills established at New Lanark by David Dale, from which Robert Owen took the model of his famous industrial village; the story of the Manchester Infirmary of 1796, when a "House of Recovery" was established; the establishment of a sort of industrial school in Birmingham; the favourable opinion expressed in 1806 by the physicians of Liverpool with regard to "vaccine inoculation"; and the improved cooking stove introduced by Count Rumford for use in the Foundling Hospital. It will thus be seen that there is plenty of variety in the book, and the story of the work done for the poor by generous and enlightened men during the time of the great war with France may well be studied by reformers of our own day.

* "Self-Help a Hundred Years Ago." By George Jacob Holyoake, author of "The History of Co-operation in England." London: Swan Sonnenschein, and Co.

